# Longitudinal Adaptive Functioning Outcomes in Children with Autism Spectrum Disorder During a 180 Day Open-Label Extension of K11-Tmax, a Consortium Probiotic Mix with Multivitamins

**DOI:** 10.3390/life16060940

**Published:** 2026-06-02

**Authors:** Deivis O. Guimaraes, Racire S. Silva, Lara A. Ferreira, Larissa Martinelli, Rebeca M. M. Werly, Raphaela F. Amorim, Lívia B. S. S. Holzbach, Roberto Badaró, Alex A. B. Santos, Elisardo C. Vasquez, Sarha A. L. de Queiroz

**Affiliations:** 1 Gon1 Biotech, Vitória City 29050-335, Espirito Santo State, Brazil; raphaela.figueira@hotmail.com (R.F.A.); liviabrunas@gmail.com (L.B.S.S.H.); 2 PPG-GETEC: Industrial Management and Technology, Senai Cimatec University, Salvador 41650-010, Bahia, Brazil; rbadaro884@gmail.com (R.B.); alex.santos@fieb.org.br (A.A.B.S.); 3 Translational Physiology and Pharmacology Laboratory, Graduate Program in Pharmaceutical Sciences, Vila Velha University, Vila Velha 29102-920, Espirito Santo State, Brazil; raciresampaio@gmail.com (R.S.S.); laraalmeidaf20@gmail.com (L.A.F.); martinelli_larissa@hotmail.com (L.M.); rebecawmotta@gmail.com (R.M.M.W.); evasquez@terra.com.br (E.C.V.); sarha.andrade@hotmail.com (S.A.L.d.Q.)

**Keywords:** autism spectrum disorder, probiotics, kefir, gut–brain axis, adaptive behavior, Vineland-3, longitudinal study, open-label extension

## Abstract

Autism spectrum disorder (ASD) is frequently associated with gastrointestinal symptoms, immune dysregulation, and altered microbiota-related signaling, supporting interest in microbiota-targeted interventions. This study evaluated adaptive functioning for over 180 days in children with ASD in a previously randomized controlled trial (RCT). After the initial 90-day blinded phase, the study continued as an open-label extension (OLE) in which all participants received K11 TMAX, a kefir-derived probiotic consortium combined with a microencapsulated micronutrient blend. The study included 130 children (3–11 years of age) who continued from the RCT were followed-up in three different trajectories: (1) placebo → K11-TMAX; (2) K11 → K11-TMAX; and (3) continued K11-TMAX supplementation. Children’s adaptive functioning was assessed by the Vineland Adaptive Behavior Scales, Third Edition (Vineland-3), Adaptive Behavior Composite (ABC) as well as by four core domains: (1) communication; (2) daily living skills; (3) socialization; and (4) motor skills. All three groups of children improved significantly on all of the parameters that were assessed with the effect sizes ranging from 0.13 to 0.43. The greatest improvement in the communication domain was seen in the transition group (1) and the greatest decrease in the externalizing behavior scores were seen in the continuous group (3) of children. Children’s adaptive functioning improved in clinically meaningful ways. Children’s improvement, however, was within the disability range and did not reach the level of typical development of children of the same age. These findings suggest supplementary therapeutic use of K11-TMAX, modulating the gut–brain axis, in children with autism spectrum disorder (ASD).

## 1. Introduction

Autism spectrum disorder (ASD) is a heterogeneous neurodevelopmental condition characterized by persistent deficits in social communication and interaction, accompanied by restricted and repetitive patterns of behavior [1]. Although ASD is clinically defined by behavioral criteria, it is increasingly recognized as a multisystem condition frequently associated with gastrointestinal disturbances, metabolic alterations, sleep problems, and immune dysregulation, all of which may contribute to symptom burden and adaptive impairment [2,3].

Within this broader biological framework, the microbiota–gut–brain axis has emerged as a particularly relevant pathway for understanding part of ASD pathophysiology [3]. This bidirectional communication system links the central nervous system to the gastrointestinal tract through neural, endocrine, immune, and metabolic signaling, allowing intestinal microbial activity to influence neurobiological processes such as neurotransmission, inflammatory tone, stress responsivity, and synaptic regulation [4,5]. In ASD, accumulating evidence suggests that alterations in gut microbial ecology may be associated with gastrointestinal dysfunction, low-grade systemic inflammation, and neurobehavioral manifestations, supporting the hypothesis that gut-associated mechanisms may be clinically relevant in at least a subgroup of affected individuals [3,6,7].

Although no single microbial signature has been consistently established in ASD, several studies have described recurrent imbalances between potentially beneficial commensals—such as *Lactobacillus* and *Bifidobacterium*—and taxa or opportunistic microorganisms more frequently associated with dysbiosis, including *Escherichia coli*, *Ruminococcus*, *Clostridium* spp., and *Candida* spp. [6,7,8,9]. These shifts may alter the intestinal metabolic environment, including the production of short-chain fatty acids (SCFAs), such as butyrate, acetate, and propionate, which are involved in epithelial homeostasis, immune signaling, and gut–brain communication [10]. Among these metabolites, butyrate is particularly relevant because of its role in maintaining epithelial integrity and supporting intestinal barrier function [10].

**Figure 1:**
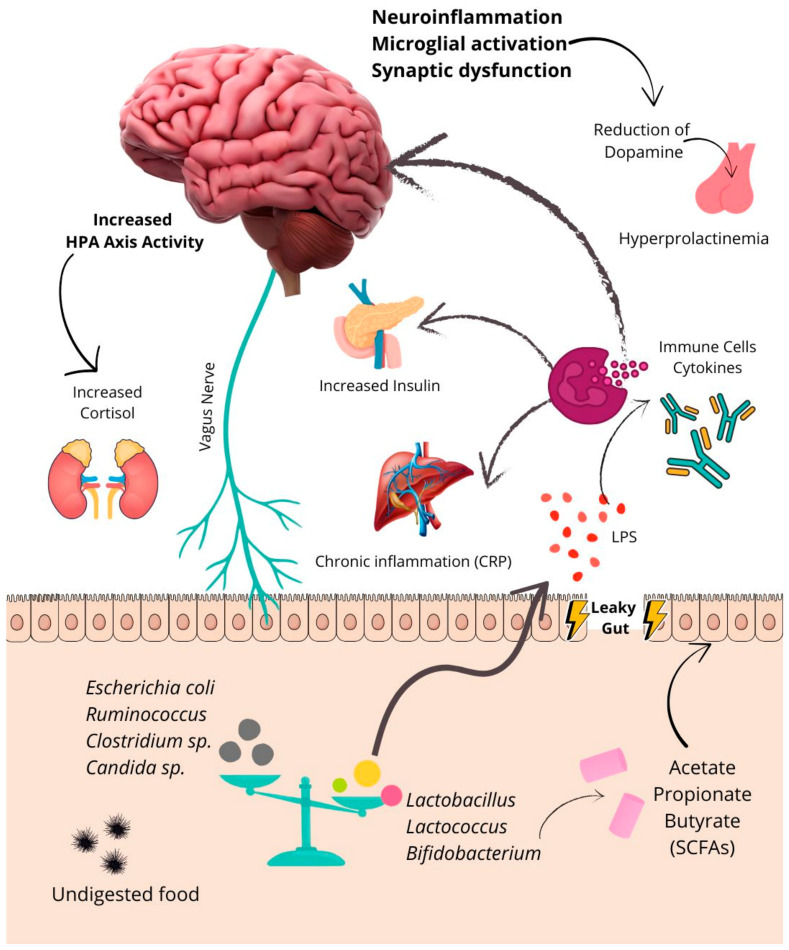
Schematic overview of the microbiota–gut–brain axis in autism spectrum disorder. The figure illustrates proposed interactions between intestinal dysbiosis, altered short-chain fatty acid (SCFA) production, increased intestinal permeability, translocation of lipopolysaccharides (LPS), immune activation, chronic inflammation, metabolic dysregulation, hypothalamic–pituitary–adrenal (HPA) axis activation, and neurobiological changes potentially relevant to autism spectrum disorder, including microglial activation, synaptic dysfunction, and altered neurotransmission.

Disruption of intestinal homeostasis may contribute to increased gut permeability, often described as “leaky gut”, facilitating the translocation of bacterial products such as lipopolysaccharides (LPS) and other pro-inflammatory mediators into systemic circulation [11,12]. These processes may amplify peripheral immune activation and, through humoral, neural, and cellular pathways, influence central inflammatory signaling and neurobiological function [12,13]. Experimental and neuropathological evidence has further supported this model by reporting altered blood–brain barrier-related proteins and increased microglial activation in ASD, findings consistent with a broader hypothesis of persistent neuroimmune dysregulation [13,14]. A schematic overview of these proposed interactions is presented in Figure 1.

Despite the biological plausibility of the gut–brain axis model in ASD, important uncertainties remain. It is not yet fully established whether microbiota-related findings represent causal mechanisms, downstream correlates, developmental adaptations, or biologically meaningful endophenotypes [7,15]. Accordingly, the therapeutic implications of these observations remain under active investigation. Probiotics have emerged as potential adjunctive interventions due to their capacity to modulate microbial composition, intestinal barrier integrity, immune signaling, and metabolite production [14,15,16]. However, the current clinical literature in ASD remains heterogeneous and methodologically constrained, with substantial variability in probiotic strains, formulations, treatment duration, and outcome measures. Notably, several studies have reported within-group improvements over time without consistent superiority over placebo, reinforcing the need for cautious interpretation and more rigorous longitudinal investigation [17,18].

Recent evidence from randomized trials, pilot studies, and systematic reviews has further highlighted both the potential and the limitations of microbiota-targeted interventions in ASD. While some studies have reported improvements in social behavior, adaptive functioning, gastrointestinal symptoms, or caregiver-reported outcomes, results remain heterogeneous and often limited by small sample sizes, methodological variability, and differences in probiotic composition and treatment duration [19,20,21,22,23,24,25,26,27,28].

A further limitation of the field is the predominance of short-term intervention designs. Most probiotic trials in ASD have evaluated relatively brief treatment periods, limiting the ability to determine whether observed effects are transient, sustained, or progressively modified over time [17,18,29]. This limitation is particularly relevant for interventions hypothesized to act through ecological and immunometabolic modulation rather than immediate pharmacological mechanisms. In such contexts, extended follow-up may be necessary to capture clinically meaningful changes in adaptive and behavioral trajectories.

In a previously conducted randomized, double blind, placebo-controlled clinical trial, kefir-derived formulations, K11 and K11 TMAX, were evaluated in children with ASD during a 90-day intervention period [17]. That study identified statistically significant within-group changes in adaptive, behavioral, inflammatory, metabolic, and microbiological outcomes, although these changes were not consistently superior to placebo in between-group analyses. Accordingly, the findings from the randomized phase supported continued investigation of the formulation while also underscoring the need for cautious interpretation regarding efficacy [17].

An important unresolved question concerns the temporal evolution of adaptive functioning beyond the duration of short-term controlled interventions. In neurodevelopmental conditions such as ASD, longitudinal changes may be nonlinear and influenced by developmental, behavioral, and contextual factors. Extended follow-up may therefore help determine whether changes observed during an initial intervention period remain stable, diminish, or continue to evolve over time. Within this framework, the present study aimed to describe adaptive functioning trajectories over 180 days in children with ASD following exposure to K11 TMAX during an open-label extension phase. The objective was not to establish confirmatory efficacy, but rather to characterize longer term patterns of functional change and provide data to inform future controlled studies with extended follow-up and integrated biological assessment.

## 2. Materials and Methods

### 2.1. Study Design and Setting

This study was designed as a longitudinal, 180-day, open-label extension of a prior 90-day double-blind RCT [17]. In the original trial, participants were allocated in a 1:1:1 ratio to placebo, K11, or K11-TMAX and followed during the initial Day 0 to Day 90 blinded phase. Details regarding sample size calculation, recruitment, eligibility criteria, randomization, blinding, and the intervention protocol during the randomized phase have been described elsewhere [17].

After completion of the blinded phase and collection of all Day 90 outcomes, the allocation code was broken and the study proceeded as an open-label extension from Day 90 to Day 180. During this extension phase, all participants received the K11-TMAX formulation. The primary objective of the extension was to evaluate the temporal evolution and sustainability of adaptive functioning outcomes under continued probiotic exposure, rather than to assess confirmatory between-group efficacy.

Participants remained analytically stratified according to their original randomized allocation, generating three longitudinal exposure trajectories:

- original placebo → K11-TMAX, corresponding to 90 days of K11-TMAX exposure during the extension phase.
- original K11 → K11-TMAX, corresponding to 90 days of K11-TMAX exposure during the extension phase.
- original K11-TMAX → K11-TMAX, corresponding to 180 days of continuous K11-TMAX exposure.

This approach allowed assessment of adaptive behavior changes after transition to the enriched formulation as well as the persistence of outcomes among those continuously exposed to K11-TMAX.

The study was conducted in a child-friendly outpatient clinical setting specifically organized to reduce sensory and behavioral distress during evaluations. Clinical follow-up and monitoring were performed by a multidisciplinary team composed of pediatricians, neuropsychologists, and a psychiatrist, following the same infrastructure and operational procedures established during the randomized phase [17].

### 2.2. Participants

Participants included in the present analysis were drawn exclusively from the sample enrolled in the original randomized clinical trial [17]. In brief, children aged 3 to 11 years with a confirmed clinical diagnosis of ASD, established according to the *Diagnostic and Statistical Manual of Mental Disorders, Fifth Edition, Text Revision* (DSM-5-TR), were eligible for the initial trial. Detailed inclusion and exclusion criteria, as well as screening and recruitment procedures, have been fully described elsewhere [17].

A total of 502 complete screening responses were initially obtained, of which 207 children met eligibility criteria and were randomized in equal allocation to placebo, K11, or K11-TMAX (*n* = 69 per group). Participants were male (82.5%) and female (17.5%), corresponding to a male-to-female ratio of approximately 4.7:1. At the end of the blinded phase (Day 90), 57 participants in the original placebo group, 65 participants in the original K11 group, and 60 participants in the original K11-TMAX group completed the first phase and were considered eligible to enter the extension period.

**Figure 2:**
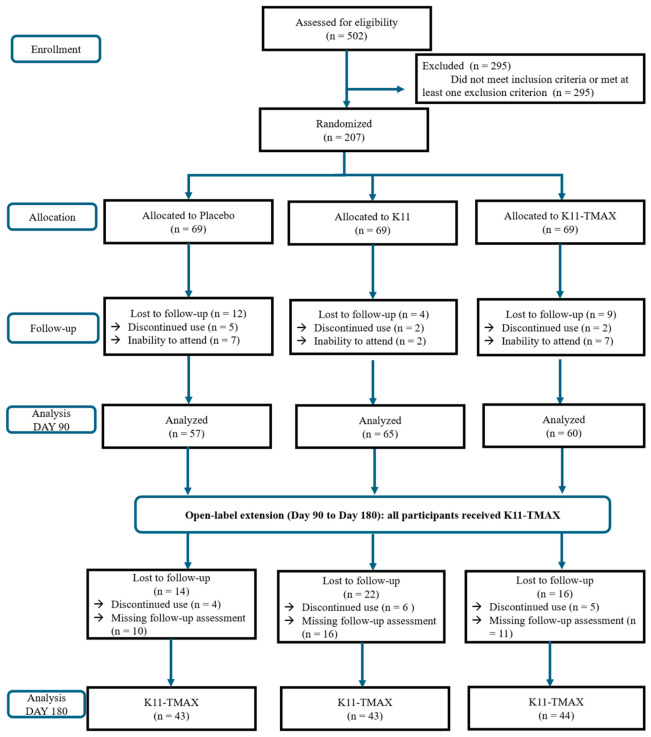
Flow diagram of participant enrollment, allocation, follow-up, and analysis. A total of 502 children were assessed for eligibility, of whom 207 were randomized as placebo, K11, or K11-TMAX (*n* = 69 per group). After the 90-day randomized phase, all participants entered an open-label extension and received K11-TMAX. Day 180 analyses were stratified according to original randomized allocation: placebo → K11-TMAX, K11 → K11-TMAX, and K11-TMAX → K11-TMAX.

For the present 180-day follow-up analysis, participants were maintained in their original randomized groups for analytical stratification, allowing evaluation of longitudinal trajectories according to prior treatment exposure. The OLE sample consisted of 130 children, corresponding to 43 participants originally allocated to placebo, 43 originally allocated to K11, and 44 originally allocated to K11-TMAX. Participants were male (82.3%) and female (17.7%), corresponding to a male-to-female ratio of approximately 4.7:1. Participant flow across both the randomized and extension phases is presented in Figure 2.

### 2.3. Intervention and Follow-Up Procedures

Each daily dose was provided as an aluminum-sealed sachet containing 300 mg of lyophilized probiotic powder, corresponding to approximately 3.0 × 10^8^ CFU/day of bacteria and 4.5 × 10^2^ CFU/day of yeasts, according to the microbiological certificate of analysis for the study batch (Batch No. 01). The safety of K11 and K11-TMAX supplementation was confirmed by toxicity and pathogenicity tests conducted by Biohall Research and Innovation (report no. ORCBH00325).

The probiotic consortium (K11) consisted of *Acetobacter orientalis* (2.37 × 10^8^ CFU), *Lactococcus* sp. (3.27 × 10^7^ CFU), *Acetobacter* sp. (1.77 × 10^7^ CFU), *Lactobacillus kefiranofaciens* (1.65 × 10^7^ CFU), *Lactobacillus kefiri* (8.10 × 10^6^ CFU), and *Kazachstania humatica* (4.47 × 10^2^ CFU). The K11-TMAX formulation contained the same probiotic base plus a taste-free microencapsulated micronutrient blend developed for pediatric use, comprising zinc (10 mg as zinc gluconate), selenium (40 μg as selenomethionine), magnesium (65 mg as magnesium citrate), and vitamin A (300 μg RAE as retinyl palmitate). These doses complied with the age-specific limits established by the Brazilian Health Regulatory Agency (ANVISA; RDC 243/2018).

Caregivers were instructed to dissolve one sachet daily in water or in the child’s usual juice and administer it orally, preferably at the same time each day, following the same preparation and administration procedures adopted during the randomized phase [29]. Standardized written and verbal instructions regarding storage, handling, and administration were reinforced at the beginning of the extension period.

Adherence during follow-up was monitored through caregiver-recorded daily intake logs, weekly telephone contact, and review of returned sachets during scheduled visits, consistent with the protocol used in the original trial [29]. Caregivers were also instructed to report any adverse events spontaneously at any time during follow-up.

Throughout the study, participants were allowed to continue regular therapies and stable pharmacological treatments, and any relevant changes in concomitant care were recorded. Families were informed that the study product should be considered an adjunctive intervention, rather than a substitute for standard clinical management.

### 2.4. Clinical Outcomes

Adaptive functioning was the primary clinical focus of the open-label extension phase and was assessed using the Vineland-3, the same instrument adopted as the primary outcome measure in the initial randomized phase [29]. Vineland-3 is a widely used measure of adaptive functioning and provides a clinically meaningful assessment of everyday performance in children with ASD. The Adaptive Behavior Composite (ABC) represents a global index of functional autonomy and is considered a clinically relevant endpoint for evaluating outcomes in this population.

Given the objectives of the extension phase, Vineland-3 was maintained as the sole prespecified clinical outcome instrument, ensuring continuity with the primary endpoint of the randomized phase and enabling a focused longitudinal assessment of functional trajectories over time. Importantly, both the primary and secondary clinical outcomes in the present analysis were derived from the same instrument, avoiding the introduction of additional measurement variability across different tools during the follow-up period.

#### 2.4.1. Administration and Scoring

Vineland-3 was administered to parents or primary caregivers using a structured interview format, conducted in accordance with the procedures described in the original randomized trial [29]. The instrument evaluates adaptive functioning across four domains: communication, daily living skills, socialization, and motor skills, and includes indices of maladaptive behavior, comprising internalizing behavior and externalizing behavior. Items were scored using a 0–2 scale (0 = never, 1 = sometimes, 2 = often), and raw scores were converted into standardized scores according to the Vineland-3 manual, allowing comparisons across individuals with different ages and developmental profiles.

For the adaptive functioning domains and the ABC, higher standardized scores indicate better functional performance and greater autonomy. Therefore, increases in these scores over time were interpreted as clinical improvement. For the maladaptive behavior domain, which includes internalizing (e.g., anxiety, withdrawal) and externalizing (e.g., irritability, aggression) behaviors, the interpretation follows the inverse direction: higher scores reflect greater severity and frequency of behavioral difficulties. Accordingly, reductions in these scores over time were interpreted as clinical improvement.

#### 2.4.2. Primary and Secondary Endpoints

The primary endpoint of the extension phase was the change in the ABC score over time. Secondary endpoints included changes in the individual Vineland-3 domain scores: communication, daily living skills, socialization, and motor skills. Additional secondary outcomes included changes in internalizing behavior and externalizing behavior scores, derived from the maladaptive behavior domain of the same instrument.

Following unblinding at Day 90, all participants received the K11-TMAX formulation. For analytical purposes, longitudinal changes were examined according to original randomized allocation, allowing assessment of adaptive functioning across distinct exposure trajectories. This framework enabled the assessment of both the emergence of adaptive changes after transition to K11-TMAX and the maintenance of functional outcomes under prolonged continuous use of the formulation.

Biological outcomes assessed during the initial randomized phase have been previously reported [29] and were not reassessed as primary endpoints during the extension phase. The decision to focus exclusively on adaptive functioning was based on the objective of characterizing clinically meaningful longitudinal changes under continued exposure, while reducing procedural burden and preserving participant retention in a pediatric ASD sample. This approach is consistent with the pragmatic nature of the extension phase and with the prioritization of patient-centered functional outcomes.

### 2.5. Statistical Analysis

Quantitative variables were summarized as means ± standard deviations, SD, and categorical data were described using absolute and relative frequencies, when applicable. Distributional assumptions were evaluated using the Kolmogorov–Smirnov test and visual inspection of the data. For within-group comparisons between time points, paired Student’s t-tests were used when the data were considered approximately normally distributed, whereas the Wilcoxon signed rank test was applied for nonnormal distributions. In addition to assessing statistical significance, the amount of change for each exposure trajectory was calculated using Cohen’s d with related 95% confidence intervals for each timepoint.

Because the Day 90 to Day 180 phase was conducted as an open-label extension in which all participants received the same formulation, K11 TMAX, the present analyses were prespecified as exploratory within-trajectory analyses rather than confirmatory between-group comparisons. Participants remained analytically stratified according to original randomized allocation, generating the following exposure trajectories: original placebo to K11 TMAX, original K11 to K11 TMAX, and original K11 TMAX to continued K11 TMAX.

For participants originally allocated to placebo or K11, follow-up values for the extension analysis corresponded to the assessment obtained after 90 days of K11 TMAX exposure during the open-label phase. For participants originally allocated to K11 TMAX, follow-up values corresponded to the Day 180 assessment after continuous exposure. Accordingly, the analyses were designed to describe longitudinal patterns of change within each exposure trajectory and should not be interpreted as evidence of comparative efficacy across groups.

Given the exploratory nature of the extension phase, no formal adjustment for multiple comparisons was applied. Therefore, *p* values should be interpreted descriptively and in conjunction with the magnitude and consistency of observed changes across domains, rather than as standalone confirmatory evidence. All analyses were performed using SPSS version 26.0, IBM Corp., Armonk, NY, USA, and GraphPad Prism version 10.2.3 (GraphPad Software, Boston, MA, USA). A two-sided significance threshold of *p* < 0.05 was adopted.

### 2.6. Ethical Approval and Consent to Participate

This open-label clinical trial was conducted in accordance with the principles of the Declaration of Helsinki and approved by the Research Ethics Committee of the University of Vila Velha (CEP/UVV), Vila Velha, ES, Brazil (CAAE No. 77402523.6.0000.5064; Opinion No. 6.715.143). Ethical approval was obtained prior to study initiation to ensure full transparency and adherence to international and national research guidelines.

Clinical trial registration: Gov.br: CAAE 77402523.6.0000.5064.

Registry name: Plataforma Brasil. Registration date: 7 February 2024.

ClinicalTrials.gov Identifier: NCT06382909.

Registry name: ClinicalTrials.gov. Registration date: 28 March 2024.

## 3. Results

### 3.1. Baseline Demographics

A total of 502 children were assessed for eligibility, of whom 207 met inclusion criteria and were randomized to placebo, K11, or K11-TMAX (*n* = 69 per group), as previously reported [17]. At the end of the randomized phase (Day 90), 57 participants in the placebo group, 65 in the K11 group, and 60 in the K11-TMAX group completed the initial follow-up and were eligible to enter the extension phase.

During the open-label follow-up (Day 90 to Day 180), additional losses occurred due to discontinuation of the intervention or missing follow-up assessments. The final sample included in the 180-day analysis comprised 130 children, corresponding to 43 participants originally allocated to placebo, 43 to K11, and 44 to K11-TMAX. Of these, 107 were boys (82.3%) and 23 were girls (17.7%), yielding a male-to-female ratio of approximately 4.7:1. This imbalance is consistent with the higher prevalence of ASD among males reported in epidemiological studies [28]. Although the exact mechanisms remain under investigation, the leading hypothesis, known as the Female Protective Effect, suggests that females may require a greater cumulative genetic burden to manifest ASD, which could contribute to the lower observed prevalence in girls.

Exploratory analyses stratified by sex revealed improvements in adaptive functioning in both male and female participants, with no statistically significant sex-by-time interaction effects. Analyses stratified by age showed mean ABC improvements of 4.2 points in children aged 3–5 years, 3.8 points in those aged 6–8 years, and 3.1 points in those aged 9–11 years. Participant flow across the randomized and extension phases is presented in Figure 2.

The analyzed sample consisted of children aged 3–11 years, with a predominance of male participants across all groups (placebo → K11-TMAX: 88%; K11 → K11-TMAX: 79%; K11-TMAX → K11-TMAX: 80%). Baseline characteristics were comparable across groups, including age (6.42 ± 2.39, 6.16 ± 2.18, and 7.12 ± 2.39 years, respectively), body mass index (BMI) (17.78 ± 4.48, 18.06 ± 5.44, and 18.98 ± 5.03 kg/m^2^), time since diagnosis (3.01 ± 1.84, 2.95 ± 1.97, and 3.04 ± 1.49 years), and autism support level (2.07 ± 0.91, 1.87 ± 0.86, and 2.19 ± 0.82) (Table 1).

Comorbidities were similarly distributed, with attention deficit hyperactivity disorder (ADHD) present in approximately 20–26% of participants across groups and generalized anxiety disorder (GAD) reported in a small proportion of cases. The use of psychopharmacological treatments—including risperidone, aripiprazole, cannabidiol, methylphenidate, and selective serotonin reuptake inhibitors (SSRIs)—was observed across all groups, without relevant differences in distribution. Notably, K11-TMAX was well tolerated, with no adverse events or treatment-related discontinuations reported during the 180-day follow-up.

### 3.2. Clinical Outcomes

Exploratory within-trajectory analyses showed statistically significant changes over time in adaptive functioning measures across the three exposure trajectories evaluated during follow-up, Table 2. Because all participants received K11 TMAX during the open-label extension, these findings are presented as longitudinal descriptive outcomes within each original allocation stratum and should not be interpreted as confirmatory between-group comparisons.

#### 3.2.1. Adaptive Behavior Composite

Participants originally allocated to placebo showed an increase in Adaptive Behavior Composite, ABC, scores after 90 days of K11 TMAX exposure during the extension phase, from 70.44 ± 10.71 to 74.74 ± 14.55, *p* < 0.0001. Participants originally assigned to K11 also showed an increase in ABC scores following transition to K11 TMAX, from 73.67 ± 12.40 to 76.72 ± 13.58, *p* = 0.0011. In participants with continuous exposure to K11 TMAX, ABC scores increased from 69.06 ± 12.27 at baseline to 73.11 ± 16.28 at Day 180, *p* < 0.0001. Collectively, these findings indicate statistically significant longitudinal changes in adaptive functioning across all three exposure trajectories.

Cohen’s d values for the within-trajectory ABC changes were d = 0.34 (95% CI: −0.09 to 0.76) for the placebo → K11-TMAX group, d = 0.23 (95% CI: −0.19 to 0.66) for the K11 → K11-TMAX group, and d = 0.28 (95% CI: −0.14 to 0.70) for the continuous K11-TMAX group.

#### 3.2.2. Core Adaptive Domains

Across all exposure trajectories, significant increases were observed in core adaptive domains, including communication, daily living skills, socialization and motor skills. In participants transitioning from placebo to K11-TMAX, communication scores increased from 68.42 ± 16.09 to 75.14 ± 19.77 (*p* < 0.00001), with parallel improvements in daily living skills (73.51 ± 12.18 to 76.93 ± 14.66; *p* = 0.0224) and socialization (71.91 ± 12.25 to 74.79 ± 14.72; *p* = 0.0200).

A similar trajectory was observed in the K11 → K11-TMAX group, with increases in communication (73.74 ± 18.47 to 77.72 ± 20.13; *p* = 0.0024), daily living skills (75.84 ± 12.01 to 78.49 ± 12.22; *p* = 0.0120), and socialization (75.37 ± 13.33 to 77.72 ± 12.56; *p* = 0.0260). In the continuous K11-TMAX group, improvements were also observed over 180 days, particularly in communication (65.04 ± 18.05 to 70.14 ± 21.91; *p* < 0.0001) and daily living skills (72.35 ± 15.05 to 77.50 ± 18.08; *p* = 0.00008), with a smaller but statistically significant increase in socialization (70.98 ± 13.47 to 72.93 ± 15.81; *p* = 0.027).

Motor skills scores showed modest but statistically significant increases across all groups. Improvements were observed in the placebo → K11-TMAX group (77.40 ± 10.88 to 80.71 ± 11.52; *p* = 0.0255), in the K11 → K11-TMAX group (79.08 ± 15.49 to 81.93 ± 19.41; *p* = 0.0462), and in the continuous K11-TMAX group (75.26 ± 14.10 to 77.71 ± 18.61; *p* = 0.0449).

Effect sizes for communication ranged from d = 0.21 to d = 0.37 across the three trajectory groups; for daily living skills, from d = 0.22 to d = 0.31; for socialization, from d = 0.13 to d = 0.21; and for motor skills, from d = 0.15 to d = 0.30. The transition from placebo to active treatment (placebo → K11-TMAX) yielded the most robust gains in Communication (d = 0.37).

#### 3.2.3. Maladaptive Behavior

Within-trajectory reductions in maladaptive behavior scores were observed over time. Internalizing behavior scores decreased significantly over time across all exposure trajectories, indicating a reduction in symptoms such as anxiety and depression. In the placebo → K11-TMAX group, scores decreased from 19.50 ± 2.42 to 18.88 ± 2.59 (*p* = 0.0059). Similar reductions were observed in the K11 → K11-TMAX group (19.77 ± 1.56 to 19.33 ± 2.14; *p* = 0.0484) and in the continuous K11-TMAX group (19.98 ± 1.95 to 19.18 ± 2.70; *p* = 0.0007).

Externalizing behavior scores also decreased significantly across all groups, reflecting reductions in irritability and aggressive behaviors. In the placebo → K11-TMAX group, scores decreased from 18.91 ± 2.35 to 18.30 ± 2.77 (*p* = 0.0429). In the K11 → K11-TMAX group, scores decreased from 18.31 ± 2.10 to 17.81 ± 2.42 (*p* = 0.0455), and in the continuous K11-TMAX group, from 18.51 ± 2.25 to 17.41 ± 2.88 (*p* = 0.0125).

Cohen’s d values for internalizing behavior reductions ranged from −0.24 to −0.34 across the three trajectory groups, while externalizing behavior reductions ranged from −0.22 to −0.43. The largest reduction was observed in the continuous K11-TMAX group for externalizing behavior (d = −0.43; 95% CI: −0.85 to −0.01).

Overall, the results demonstrate a consistent pattern of longitudinal improvement in adaptive functioning and behavioral regulation across all exposure trajectories, including participants who transitioned from placebo or K11 to K11-TMAX and those with continuous exposure over 180 days. These findings indicate coherent within-subject changes over time, with parallel improvements in adaptive domains and reductions in maladaptive behaviors.

## 4. Discussion

The gains in adaptive functioning observed over the 90-day trial period were sustained and, in some areas, continued to improve over the further 180 days. Children who had been on placebo throughout the trial and then went on to K11-TMAX showed improvements in most areas. The children who had been on K11-TMAX throughout the trial maintained and continued to improve in many areas. It is important to note that the mean scores for all three groups remained below that of the general population at all time points. Thus, the children in the study had made gains in their daily living skills within the disability range but were not yet functioning like non-disabled children. A 5- to 7-point increase in daily living skills would translate to a child being able to complete more daily living tasks without assistance (e.g., dressing, feeding self, hygiene). A 6- to 8-point decrease in internalizing behavior would translate to a child displaying less anxious and withdrawn behaviors. Importantly, these are the types of changes that families report as meaningful in anecdotal reports. These types of functional and behavioral changes may be particularly relevant for families, as caregiver burden and family resilience are important dimensions of autism spectrum disorder management [30,31].

Effect sizes for the various measures ranged from d = 0.13 to d = 0.43. Given the exploratory nature of this open-label study, these are modest findings and require replication. Communication showed the largest improvement in the group that had just initiated K11-TMAX (d = 0.37). Externalizing behavior showed the largest reduction in the group with the longest exposure to K11-TMAX (d = 0.43). Once again, these are signal-detection findings and require confirmation in a controlled trial.

A further question is whether the changes in gut microbiome in this study’s participants are causal in ASD or are exacerbating. This study was not designed to answer this question and it would require a controlled trial. However, our findings are in agreement with previous work [17], where an increase in *Lactobacillus* spp. and a decrease in *Escherichia coli* in conjunction with decreased levels of inflammatory markers (CRP, cortisol, fecal calprotectin) was noted following intervention with K11-TMAX. These findings are in line with a bidirectional gut–brain axis model where the gut microbiome influences the brain and behavior but in the case of individuals with ASD, the gut microbiome may be disturbed and in a state of dysbiosis which in turn exacerbates behavioral symptoms.

Several limitations of this study must be noted. The first is that this was an open-label study which means that all participants and their families knew what intervention the child was receiving throughout the study. This may introduce reporting bias into the results. A parallel placebo group was not included in this study. The participants in this study were predominantly male (82.3%, approximately 4.7 to 1). Thus, the findings in this study are limited to males with ASD and would not apply to females with ASD. In addition, the sample size in this study was too small to perform any meaningful sex-stratified analyses. The exploratory subgroup analyses performed for age were restricted by sample size and the results would not be reliable in any of the three age-bands (3 to 5 years, 6 to 8 years, 9 to 11 years) within each stratum. In a review of the manuscript, the authors noted that there were potential age-related differences in dietary intake as well as in gut microbiome composition that would be of interest in future studies.

Taken together, the present findings indicate consistent longitudinal patterns of improvement in adaptive functioning and behavioral regulation in children with autism spectrum disorder following exposure to K11 TMAX over 180 days. Although the open-label design does not allow causal inference, the coherence of changes observed across multiple Vineland-3 domains provides meaningful longitudinal signals in a field still marked by heterogeneity in probiotic formulations, study duration, and outcome assessment. These findings add to the growing literature on microbiota-targeted strategies in neurodevelopmental disorders and offer a structured starting point for future work. In this context, the relevance of extended follow-up is further reflected in the approval by the Brazilian National Research Ethics Commission, CONEP, of a subsequent large scale longitudinal study involving 1500 children, which will offer an opportunity to further examine the durability, clinical consistency, and broader applicability of these findings.

## 5. Conclusions

This longitudinal extension clinical trial achieved its objective of characterizing the temporal trajectory of adaptive functioning in children with autism spectrum disorder following sustained exposure to K11 TMAX, a kefir-derived formulation enriched with micronutrients. The findings showed consistent improvements over a 180-day period in adaptive functioning and behavioral regulation, including among participants who transitioned from placebo or K11 to K11 TMAX and among those who remained under continuous exposure.

The coherence of these longitudinal patterns across multiple Vineland-3 domains, together with parallel reductions in maladaptive behaviors, supports the presence of structured and clinically relevant functional signals over time. In this context, the open-label extension provided an important temporal perspective beyond the initial randomized phase, allowing a broader characterization of adaptive changes during continued follow-up and contributing to a more comprehensive understanding of response trajectories in this population.

From an academic and translational standpoint, this study contributes to the growing field of ASD research by adding a structured longitudinal clinical trial data set. The study investigated the tolerability and feasibility of a multicomponent approach addressing both the microbial and the micronutrient aspects in children with ASD.

Overall, these findings contribute to the advancement of microbiota-targeted interventions in ASD by underscoring the value of extended follow-up and temporally informed outcome assessment. They also provide an empirical basis for future larger-scale and mechanistically informed studies aimed at further clarifying the durability, specificity, and clinical relevance of this intervention strategy.

## Figures and Tables

**Table 1:** Baseline characteristics of participants included in the 180-day analysis.

Parameters	Placebo → K11-TMAX, Day 90 (*n* = 43)	K11 → K11-TMAX, Day 90 (*n* = 43)	K11-TMAX → K11-TMAX, Day 180 (*n* = 44)
Age (years)	6.42 ± 2.39	6.16 ± 2.18	7.12 ± 2.39
Gender			
Male	88%	79%	80%
Female	12%	21%	20%
BMI (kg/m^2^)	17.78 ± 4.48	18.06 ± 5.44	18.98 ± 5.03
Time of diagnosis (years)	3.01 ± 1.84	2.95 ± 1.97	3.04 ± 1.49
Support Level	2.07 ± 0.91	1.87 ± 0.86	2.19 ± 0.82
Level 1	37%	44%	26%
Level 2	19%	25%	29%
Level 3	44%	31%	45%
Comorbidities	ADHD 25.6%	ADHD 20% GAD 2%	ADHD 25.5% GAD 2%
Use of psychopharmaceuticals	Risperidone 16.3%	Risperidone 35.42%	Risperidone 39.21%
	Aripiprazole 18.6%	Aripiprazole 10.42%	Aripiprazole 9.8%
	Cannabidiol 6.97%	Cannabidiol 10.42%	Cannabidiol 5.88%
	Methylphenidate 11.63%	Methylphenidate 4.16%	Methylphenidate 9.8%
		SSRIs 6.25%	SSRIs 1.96%

**Table 2:** Vineland-3 scores at baseline and follow-up, according to exposure trajectories.

Vineland-3	Baseline	Follow-Up	*p*-Value	Effect Size (Cohen’s d) 95% CI
**Initial Group, Placebo**		**Day 90 with K11-TMAX (*n* = 43)**		
Communication	68.42 ± 16.09	75.14 ± 19.77	<0.00001 *	0.37
Daily Living Skills	73.51 ± 12.18	76.93 ± 14.66	0.0224 *	0.25
Socialization	71.91 ± 12.25	74.79 ± 14.72	0.0200 *	0.21
Adaptive Behavior Composite	70.44 ± 10.71	74.74 ± 14.55	<0.0001 *	0.34 [−0.09, 0.76]
Motor Skills	77.40 ± 10.88	80.71 ± 11.52	0.0255 *	0.30
Internalizing Behavior	19.50 ± 2.42	18.88 ± 2.59	0.0059 *	−0.25
Externalizing Behavior	18.91 ± 2.35	18.30 ± 2.77	0.0429 *	−0.24
**Initial Group—K11**		**Day 90 with K11-TMAX (*n* = 43)**		
Communication	73.74 ± 18.47	77.72 ± 20.13	0.0024 *	0.21
Daily Living Skills	75.84 ± 12.01	78.49 ± 12.22	0.0120 *	0.22
Socialization	75.37 ± 13.33	77.72 ± 12.56	0.0260 *	0.18
Adaptive Behavior Composite	73.67 ± 12.40	76.72 ± 13.58	0.0011 *	0.23 [−0.19, 0.66]
Motor Skills	79.08 ± 15.49	81.93 ± 19.41	0.0462 *	0.16
Internalizing Behavior	19.77 ± 1.56	19.33 ± 2.14	0.0484 *	−0.24
Externalizing Behavior	18.31 ± 2.10	17.81 ± 2.42	0.0455 *	−0.22
**Initial Group—K11-TMAX**		**Day 180 with K11-TMAX (*n* = 44)**		
Communication	65.04 ± 18.05	70.14 ± 21.91	<0.0001 *	0.25
Daily Living Skills	72.35 ± 15.05	77.50 ± 18.08	0.00008 *	0.31
Socialization	70.98 ± 13.47	72.93 ± 15.81	0.027 *	0.13
Adaptive Behavior Composite	69.06 ± 12.27	73.11 ± 16.28	0.00003 *	0.28 [−0.14, 0.70]
Motor Skills	75.26 ± 14.10	77.71 ± 18.61	0.0449 *	0.15
Internalizing Behavior	19.98 ± 1.95	19.18 ± 2.70	0.0007 *	−0.34
Externalizing Behavior	18.51 ± 2.25	17.41 ± 2.88	0.0125 *	−0.43

The quantitative data are presented as the means ± standard deviations. Values with an asterisk (*) indicate statistical significance (*p* < 0.05) compared with the baseline, as determined by the paired *t*-test when the data followed a normal distribution and by the Wilcoxon test when the data followed a nonnormal distribution. Cohen’s d values were calculated as the mean difference divided by the pooled standard deviation. Values in brackets represent the 95% confidence interval of Cohen’s d. Effect sizes of 0.2, 0.5, and 0.8 were considered small, moderate, and large, respectively.

## Data Availability

The summary data supporting the findings of this study are presented in the article. Additional deidentified data may be made available by the corresponding author upon reasonable request, subject to ethical and institutional restrictions applicable to pediatric clinical research.

## Abbreviations

The following abbreviations are used in this manuscript:

ADHD	Attention-Deficit/Hyperactivity Disorder
ANVISA	Brazilian Health Regulatory Agency (National Health Surveillance Agency)
ASD	Autism Spectrum Disorder
BMI	Body Mass Index
CFU	Colony-Forming Units
DSM 5-TR	Diagnostic and Statistical Manual of Mental Disorders, Fifth Edition, Text Revision
GAD	Generalized Anxiety Disorder
HPA	Hypothalamic–pituitary–adrenal
LPS	Lipopolysaccharides
OLE	Open-Label Extension
SCFAs	Short-chain fatty acids
SD	Standard Deviation
SSRI	Selective Serotonin Reuptake Inhibitor
Vineland-3	Vineland Adaptive Behavior Scales, Third Edition

## References

[1] American Psychiatric Association (2022). Diagnostic and Statistical Manual of Mental Disorders.

[2] Xu M., Xu X., Li J., Li F. (2019). Association between gut microbiota and autism spectrum disorder: A systematic review and meta-analysis. Front. Psychiatry.

[3] Rutsch A., Kantsjö J.B., Ronchi F. (2020). The gut–brain axis: How microbiota and host inflammasome influence brain physiology and pathology. Front. Immunol..

[4] Srikantha P., Mohajeri M.H. (2019). The possible role of the microbiota–gut–brain axis in autism spectrum disorder. Int. J. Mol. Sci..

[5] Fiorentino M., Sapone A., Senger S., Camhi S.S., Kadzielski S.M., Buie T.M., Kelly D.L., Cascella N., Fasano A. (2016). Blood–brain barrier and intestinal epithelial barrier alterations in autism spectrum disorders. Mol. Autism.

[6] Madore C., Leyrolle Q., Lacabanne C., Benmamar-Badel A., Joffre C., Nadjar A., Layé S. (2016). Neuroinflammation in autism: Plausible role of maternal inflammation, dietary omega 3, and microbiota. Neural Plast..

[7] Hsiao E.Y., McBride S.W., Hsien S., Sharon G., Hyde E.R., McCue T., Codelli J.A., Chow J., Reisman S.E., Petrosino J.F. (2013). Microbiota modulate behavioral and physiological abnormalities associated with neurodevelopmental disorders. Cell.

[8] Liu Y.W., Liong M.T., Chung Y.E., Huang H.Y., Peng W.S., Cheng Y.F., Lin Y.S., Wu Y.Y., Tsai Y.C. (2019). Effects of Lactobacillus plantarum PS128 on children with autism spectrum disorder in Taiwan: A randomized, double blind, placebo controlled trial. Nutrients.

[9] Lai Y., Dhingra R., Zhang Z., Ball L.M., Zylka M.J., Lu K. (2022). Toward elucidating the human gut microbiota–brain axis: Molecules, biochemistry, and implications for health and diseases. Biochemistry.

[10] Zhuang M., Zhang X., Cai J. (2024). Microbiota–gut–brain axis: Interplay between microbiota, barrier function and lymphatic system. Gut Microbes.

[11] Chamtouri M., Merghni A., Miranda-Cadena K., Sakly N., Gaddour N., de los Reyes-Gavilán C.G., Mastouri M., Eraso E., Quindós G. (2024). Characterization of yeast isolated from the gut microbiota of Tunisian children with autism spectrum disorder. J. Fungi.

[12] Cryan J.F., O’Riordan K.J., Cowan C.S.M., Sandhu K.V., Bastiaanssen T.F., Boehme M., Codagnone M.G., Cussotto S., Fulling C., Golubeva A.V. (2019). The microbiota–gut–brain axis. Physiol. Rev..

[13] Ng Q.X., Peters C., Ho C.Y.X., Lim D.Y., Yeo W.S. (2018). A meta-analysis of the use of probiotics to alleviate depressive symptoms. J. Affect. Disord..

[14] Fenster K., Freeburg B., Hollard C., Wong C., Laursen R.R., Ouwehand A.C. (2019). The production and delivery of probiotics: A review of a practical approach. Microorganisms.

[15] Heidebach T., Forst P., Kulozik U. (2012). Microencapsulation of probiotic cells for food applications. Crit. Rev. Food Sci. Nutr..

[16] Mohamadzadeh M., Fazeli A., Shojaosadati S.A. (2024). Polysaccharides and proteins-based bionanocomposites for microencapsulation of probiotics to improve stability and viability in the gastrointestinal tract: A review. Int. J. Biol. Macromol..

[17] de Queiroz S.A.L., Guimarães D.O., Ferreira L.A., Martinelli L., Werly R.M., Amorim R.F., Holzbach L.B., Badaró R., Santos A.A., Vasquez E.C. (2026). Kefir-derived probiotic mixture for children with autism spectrum disorder: A double blind randomized clinical trial. BMC Pediatr..

[18] Takumi T., Tamada K., Hatanaka F., Nakai N., Bolton P.F. (2020). Behavioral neuroscience of autism. Neurosci. Biobehav. Rev..

[19] Kong X.J., Liu J., Liu K., Koh M., Sherman H., Liu S., Tian R., Sukijthamapan P., Wang J., Fong M. (2021). Probiotic and oxytocin combination therapy in patients with autism spectrum disorder: A randomized, double blinded, placebo controlled pilot trial. Nutrients.

[20] Mazzone L., Dooling S.W., Volpe E., Uljarević M., Waters J.L., Sabatini A., Arturi L., Abate R., Riccioni A., Siracusano M. (2024). Precision microbial intervention improves social behavior but not autism severity: A pilot double blind randomized placebo controlled trial. Cell Host Microbe.

[21] Soleimanpour S., Abavisani M., Khoshrou A., Sahebkar A. (2024). Probiotics for autism spectrum disorder: An updated systematic review and meta-analysis of effects on symptoms. J. Psychiatr. Res..

[22] He X., Liu W., Tang F., Chen X., Song G. (2023). Effects of probiotics on autism spectrum disorder in children: A systematic review and meta-analysis of clinical trials. Nutrients.

[23] Hassib L., Kanashiro A., Pedrazzi J.F.C., Vercesi B.F., Higa S., Arruda Í., Soares Y., Souza A.d.J.d., Barichello T., Guimarães F.S. (2025). Microbiota-based therapies as novel targets for autism spectrum disorder: A systematic review and meta-analysis. Prog. Neuropsychopharmacol. Biol. Psychiatry.

[24] Wong G.C., Montgomery J.M., Taylor M.W., Grabrucker A.M. (2021). The gut–microbiota–brain axis in autism spectrum disorder. Autism Spectrum Disorders.

[25] Meidani F.Z., Rahmati R., Mostafavi M., Darvishi M., Khodadadi S., Mohammadi M., Shamlou F., Bakhtiyari S., Alipourfard I. (2024). Gut microbiota and autism spectrum disorder: A neuroinflammatory mediated mechanism of pathogenesis?. Inflammation.

[26] Iglesias-Vázquez L., Van Ginkel Riba G., Arija V., Canals J. (2020). Composition of gut microbiota in children with autism spectrum disorder: A systematic review and meta-analysis. Nutrients.

[27] Sharon G., Cruz N.J., Kang D.W., Gandal M.J., Wang B., Kim Y.M., Zink E.M., Casey C.P., Taylor B.C., Lane C.J. (2019). Human gut microbiota from autism spectrum disorder promote behavioral symptoms in mice. Cell.

[28] Loomes R., Hull L., Mandy W.P.L. (2017). What Is the Male-to-Female Ratio in Autism Spectrum Disorder? A Systematic Review and Meta-Analysis. J. Am. Acad. Child Adolesc. Psychiatry.

[29] Restoy D., Oriol-Escudé M., Alonzo-Castillo T., Magán-Maganto M., Canal-Bedia R., Díez-Villoria E., Gisbert-Gustemps L., Setién-Ramos I., Martínez-Ramírez M., Ramos-Quiroga J.A. (2024). Emotion regulation and emotion dysregulation in children and adolescents with autism spectrum disorder: A meta-analysis of evaluation and intervention studies. Clin. Psychol. Rev..

[30] van Niekerk K., Stancheva V., Smith C. (2023). Caregiver burden among caregivers of children with autism spectrum disorder. S. Afr. J. Psychiatr..

[31] Chen X., Tao J., Zhang Y., Xu Q., Dong C. (2025). Relationship between caregiver burden and family resilience among Chinese parents of children with autism spectrum disorder: The mediating role of social support and positive cognition. J. Pediatr. Nurs..

